# Preparation of Wheat Straw Hot-Pressed Board through Coupled Dilute Acid Pretreatment and Surface Modification

**DOI:** 10.3390/ma17091950

**Published:** 2024-04-23

**Authors:** Jianing Wang, Libo Zhang, Yepeng Xiao, Qinzhen Fan, Chong Yang, Yiqiang Deng, Hao Lu, Lihua Cheng

**Affiliations:** 1 Center of New Energy Research, School of Intelligence Science and Technology Xinjiang University, Urumqi 830047, China; wangjianing62022@163.com; 2 Guangdong Provincial Engineering & Technology Center for Corrosion and Safety in Petrochemical Industry, School of Chemical Engineering, Guangdong University of Petrochemical Technology, Maoming 525000, China; yepengxiao@gdupt.edu.cn (Y.X.); fanqinzhen@163.com (Q.F.); yangcgh@163.com (C.Y.); yqdeng@gdupt.edu.cn (Y.D.); 13828684794@139.com (L.C.); 3 State Key Laboratory of Heavy Oil Processing, College of Engineering, China University of Petroleum-Beijing at Karamay, Karamay 834000, China

**Keywords:** wheat straw, acid pretreatment, surface modification, hot-pressed board

## Abstract

The production of wheat straw waste board materials encounters challenges, including inadequate inherent adhesiveness and the utilization of environmentally harmful adhesives. Employing a hot-pressed method for converting wheat straw into board materials represents a positive stride towards the resourceful utilization of agricultural wastes. This study primarily focuses on examining the influence of hot-pressing process conditions on the mechanical properties of wheat straw board materials pretreated with dilute acid. Additionally, it assesses the necessity of dilute acid treatment and optimizes the hot-pressing conditions to achieve optimal results at 15 MPa, 2 h, and 160 °C. Furthermore, a comprehensive process is developed for preparing wheat straw hot-pressed board materials by combining dilute acid pretreatment with surface modification treatments, such as glutaraldehyde, citric acid, and rosin. Finally, a thorough characterization of the mechanical properties of the prepared board materials is conducted. The results indicate a substantial improvement in tensile strength across all modified wheat straw board materials compared to untreated ones. Notably, boards treated with glutaraldehyde exhibited the most significant enhancement, achieving a tensile strength of 463 kPa, bending strength of 833 kPa, and a water absorption rate of 14.14%. This study demonstrates that combining dilute acid pretreatment with surface modification treatments effectively enhances the performance of wheat straw board materials, offering a sustainable alternative to traditional wood-based board materials.

## 1. Introduction

In recent years, the heightened consciousness towards environmental preservation has sparked widespread interest in the utilization of agricultural byproducts for the preparation of eco-friendly materials. Wheat, a cereal crop with a high economic value, has emerged as one of the most widely distributed, extensively cultivated, and second-highest in total production globally, making it a prime candidate for the preparation of sustainable and environmentally friendly material [1]. In 2023, the global annual production of wheat reached 786 million tons [2]. Wheat straw, an agricultural byproduct of wheat, accounted for 66.7% of the total weight of wheat. Despite wheat straw’s abundance of cellulose, hemicellulose, and lignin, its high ash content, primarily silica, and the presence of waxes render it unsuitable as a raw material for fine chemical production. Consequently, the majority of wheat straw is discarded and burned, leading to environmental degradation and significant resource waste. 

Board materials, integral to daily construction and manufacturing activities, achieved a substantial global market value of USD 175.1 billion in 2023 [3]. Traditional wood-based boards not only consume a considerable amount of wood during production but also rely on adhesives, such as formaldehyde resins, to boost their performance strength [4]. These adhesives have the potential to release formaldehyde and other harmful substances, thereby posing significant risks to both the environment and human health [5]. Consequently, there has been a steady increase in interest for adhesive-free board research [6]. Adhesive-free boards primarily achieve their desired strength through the inherent self-adhesive properties of woody cellulose biomass. Nevertheless, the presence of minor components within wheat straw, such as waxes and pectins, hinders the compatibility and adhesion among wheat straw particles, thereby affecting the self-adhesive performance of the boards [7]. Furthermore, wheat straw possesses a rigid epidermis which is abundant in silica [8], leading to compromised self-adhesiveness in hot-pressed wheat straw boards.

Currently, researchers are utilizing various pretreatments on lignocellulosic biomasses to modify and enhance their inherent self-adhesive properties. One such pretreatment, mechanical pulverization, effectively reduces the particles size of raw material, increasing their specific surface area and ultimately improving self-adhesiveness [9]. However, mechanical pulverization does result in elevated processing costs and energy consumption, making it more commonly employed alongside other processing methods to achieve optimal results [10]. The alkali pretreatment involves treating lignocellulosic biomass with a solution of NaOH or KOH to eliminate most of its silica, lignin, and hemicellulose. This treatment leads to the collapse of hollow fibers in wheat straw and restructures the cellulose, lignin, and hemicellulose, thereby enhancing their self-adhesiveness during the hot-pressing process. Consequently, this pretreatment not only boosts the mechanical strength of the resulting boards but also optimizes their overall performance [11]. Hu et al. [12] successfully developed a highly dense wood material with an orderly alignment of cellulose nanofibers. This achievement was realized by first partially removing lignin and hemicellulose from the wood through an alkali pretreatment using NaOH and Na_2_SO_3_. Subsequently, the wood cell walls were compressed and collapsed at 10 °C, resulting in a material with superior mechanical properties and potential applications in various industries. While alkaline pretreatment can be effective, it does pose challenges such as accelerating equipment corrosion and generating waste liquid that requires costly treatment. Alternatively, acid pretreatment employs specific concentrations of acids such as hydrochloric acid, phosphoric acid, or sulfuric acid to treat lignocellulosic biomass, focusing on the removal of hemicellulose [13]. This treatment modifies the spatial arrangement of cellulose, hemicellulose, and lignin within wheat straw, subsequently enhancing the self-adhesiveness and mechanical strength of the hot-pressed boards. The dilute acid pretreatment process induces modifications in the spatial arrangement of cellulose, hemicellulose, and lignin within wheat straw. In contrast to concentrated acid and alkali treatments, dilute acid pretreatment offers safer operation and easier handling, significantly mitigating safety risks and experimental costs. Consequently, this study opts for the dilute acid pretreatment method to achieve optimal results.

Shusaku et al. [14] prepared hot-pressed boards by pretreating rice husks with hot-compressed water. However, the preparation of hot-compressed water involves intricate and laborious procedures, including thermocouple treatment, which adds complexity to the overall process. Shi et al. [15] successfully fabricated hot-pressed boards through a multistep process involving crushing bamboo, separating bamboo fibers and parenchyma cells, and mixing bamboo fibers of varying sizes. Although the resulting boards demonstrated excellent performance, the crushing step was particularly energy-intensive, especially when aiming to achieve a fineness of over 200 mesh.

With the increasing demand for sustainable biomass, Obreja et al. [16] studied the feasibility of reed as a sustainable biomass for obtaining bioenergy. Ghisman et al. [17] studied the feasibility of using sewage sludge as an important biological resource for sustainable agriculture to improve farmland soil. From the economic point of view, wheat straw is a renewable biomass resource, and the study on the use of wheat straw to prepare hot-pressed boards is conducive to sustainable management.

The objective of this study was to investigate and optimize the operational conditions for hot-pressing wheat straw into boards, to confirm that coupled dilute acid pretreatment and surface modification treatment can effectively improve the performance of hot-pressed wheat straw boards, and to explain the mechanism of various operational parameters on the formation and the mechanism of dilute acid pretreatment and surface modification treatment to improve the performance of hot-pressed wheat straw board. It discusses the effects and mechanisms of various operational parameters on the formation and performance of wheat straw boards. By employing dilute acid pretreatment followed by hot-pressing, it is observed that the strength of wheat straw boards improved significantly compared to untreated boards. Furthermore, surface modifications, including rosin, citric acid, and glutaraldehyde, are applied to dilute acid-treated wheat straw, further enhancing the physical and mechanical properties of the boards. The novelty of this study is to explore the great potential of the coupled surface modification of wheat straw on the basis of dilute acid pretreatment to prepare nonadhesive hot-pressed boards, providing an environmentally friendly and sustainable alternative to traditional biomass boards.

## 2. Materials and Methods

### 2.1. Material

Wheat straw was collected from a farm located in Urumqi, China. Initially, the wheat straw was pulverized and subsequently washed several times with water to remove the ash. Laboratory sieves were used to select particles sized between 3.0 mm and 5.0 mm, which were then dried at 105 °C for 12 h. All chemicals, including dilute hydrochloric acid (0.5 M), citric acid, glutaraldehyde (50% *w*/*v*), anhydrous ethanol, magnesium chloride hexahydrate (99%), polyvinyl alcohol, and sodium dihydrogen phosphate anhydrous (99%), were all purchased from China Innochem Chemical Reagents Co. (Beijing, China). Deionized water was produced using a WPUP-UV-20 device manufactured by Chengdu Water Science and Technology Co, Ltd. (Chengdu, China). Rosin was acquired from China SuMao Co. (Changzhou, China). All reagents were used without further purification.

### 2.2. Methods

**Figure 1:**
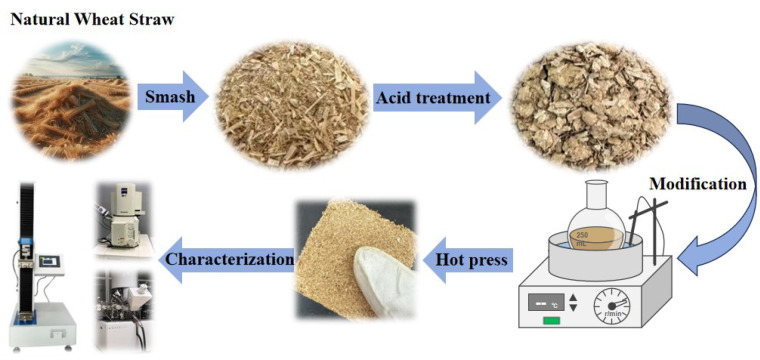
Schematic diagram of the preparation of wheat straw board by coupled dilute acid pretreatment and surface modification treatment.

The experimental process of this project is depicted in Figure 1.

#### 2.2.1. Dilute Acid Pretreatment of Wheat Straw

First, 20 g of previously dried wheat straw was placed a round-bottomed flask within 200 mL of 0.5 M dilute hydrochloric acid. Magnetic stirring at 2000 rpm was used to conform their complete mixing. The mixture was heated in a 95 °C water bath for 4 h. Afterward, we vacuum filtered the product and rinsed it with deionized water until the filtrate achieved a neutral pH value. To achieve a moisture content below 4%, we dried the obtained wheat straw in a 105 °C oven for 12 h. We stored the pretreated wheat straw in a sealed bag for subsequent use.

#### 2.2.2. Preparation of Wheat Straw Hot-Pressed Board Pretreated with Dilute Acid

Utilizing the single variable method, which means that for the three variables of hot-pressing pressure, temperature, and time, only one of the hot-pressing process conditions is changed, and the other two hot-pressing process conditions are controlled unchanged, an investigation was conducted to assess the impact of hot-pressing time, temperature, and pressure on the performance of dilute acid-pretreated wheat straw boards. Specifically, 6.5 g of dilute acid-pretreated wheat straw, which had been dried and stored in sealed bags, were weighed and placed within a hot-pressing mold measuring 50 mm × 50 mm × 5 mm. The wheat straw was then hot-pressed at various temperatures (100 °C, 120 °C, 140 °C, 160 °C, 200 °C), pressures (5 MPa, 10 MPa, 15 MPa, 20 MPa, 25 MPa, 30 MPa), and durations (1 h, 2 h, 3 h, 4 h, 5 h, 6 h) to determine the optimal hot-pressing conditions. The boards produced under these optimal conditions, utilizing dilute acid pretreatment, were designated as WSA. Conversely, boards prepared without dilute acid pretreatment, but under identical conditions, served as the control and were denoted as WS.

#### 2.2.3. Surface Modification-Citric Acid

The pretreatment methods were based on reference [18] with slight modifications. First, 20 g of dilute acid-pretreated wheat straw was soaked in a 200 mL solution of 6.9% *w*/*v* citric acid and 6.5% *w*/*v* sodium dihydrogen phosphate at room temperature and atmospheric pressure for 4 h. Following this, the product was vacuum filtered and rinsed with deionized water until the filtrate achieved a neutral pH. The filtered product was dried in an oven at 105 °C for 12 h. Subsequently, according to the optimum hot-pressing process conditions previously determined, the dried product was hot-pressed into boards. The resulting board was designated as WSA-CA.

#### 2.2.4. Surface Modification-Glutaraldehyde

The pretreatment methods were slightly modified according to reference [19]. Firstly, 20 g of acid-pretreated wheat straw was placed in 200 mL solution with polyvinyl alcohol (20% *w*/*v*) and glutaraldehyde (10% *w*/*v*) at atmospheric pressure at 40 °C for 4 h. Following this, we added 1.5% magnesium chloride hexahydrate as a catalyst during the process. Afterward, the product was vacuum filtered and rinsed with deionized water. The filtered product was dried in an oven at 120 °C for 6 h. Finally, according to the optimum hot-pressing process conditions previously determined, the dried product was hot-pressed into boards. The resulting board was designated as WSA-G.

#### 2.2.5. Surface Modification-Rosin

The pretreatment methods were based on reference [20] with minor modifications. Firstly, 20 g acid-pretreated wheat straw was soaked in a 200 mL ethanol solution of (20% *w*/*v*) rosin in ethanol at room temperature and atmospheric pressure for 4 h. Following this, the product was vacuum filtered and rinsed thoroughly with ethanol. Subsequently, the filtered product was dried in an oven at 60 °C for 6 h. Finally, according to the optimum hot-pressing process conditions previously determined, the dried product was hot-pressed into boards. The resulting board was designated as WSA-R.

### 2.3. Characterization

#### 2.3.1. Mechanics Performance Testing

Each prepared wheat straw base board was precision-cut into samples measuring 30 ± 1 × 10 ± 1 mm. Following the Chinese National Standard GB/T 11718-2009, a digital display single-arm microcontrolled electronic universal testing machine(STX1000, EAST, Xiamen, China) was employed to assess tensile strength (TS) and bending strength (BS) [20]. Five replicates were tested for each sample set, and the average values were computed to ensure reliability and accuracy [21].

#### 2.3.2. Morphology Analysis

Prior to analysis, we ensured that each set of samples was completely dry and securely fastened onto the sample stage using carbon tape. Subsequently, we sputter-coated the samples with gold under vacuum to enhance conductivity. We placed the coated samples into the chamber of an environmental scanning electron microscope (Quanta 200, FEI, Valley City, ND, USA); we set the microscope’s voltage to 15 kV and fine-tuned the imaging parameters. We magnified the view 200 times, selected an appropriate field of view, and meticulously observed the surface of the board samples, ensuring to record and securely store the captured images for future reference.

#### 2.3.3. Water Adsorption of Board

We cut the prepared wheat straw base boards samples into standard specimens with dimensions of 10 mm ± 1 mm × 10 mm ± 1 mm and measured their weight. In accordance with the Chinese standard (Wood Industry Research Institute, CAF, 2013) [10], the balanced samples were submerged in water at room temperature for a duration of 24 h. Following this, the samples were gently towel-dried to eliminate any surface water droplets. Subsequently, they were weighed, and this process was repeated for five replicates within each sample group. The average water absorption rate was then computed to ensure consistency and accuracy.

#### 2.3.4. Fourier Transform Infrared (FTIR)

Fourier transform infrared spectroscopy (FTIR) was conducted utilizing a Thermo Scientific iN10 spectrometer (Waltham, MA, USA), encompassing a wavenumber range of 600–4000 cm^−1^ for comprehensive spectral analysis [22], adopting absorbance testing mode.

#### 2.3.5. Thermogravimetric Analysis (TGA)

The thermal stability of the boards was analyzed using the thermogravimetric analysis (TGA) method. The TGA runs were conducted using the Hitachi High-Tech Science model (STA7200). The prepared wheat straw base boards samples (ca. 5 mg) were heated at a rate of 5 °C·min^−1^ under a nitrogen flow (purity > 99.9999% vol) at 300 mL·min^−1^. The test temperature range was 30–700 °C [14].

## 3. Results and Discussion

### 3.1. Effects of Operating Parameters and Dilute Acid Treatment on Properties of Wheat Straw Boards

After dilute acid pretreatment, the material structure is changed, and the optimal hot-pressing process conditions (temperature, time, and pressure) are different from the untreated hot-pressing process conditions. However, the purpose of this study was to investigate the influence of coupling dilute acid pretreatment and surface modification treatment on the performance of wheat straw hot-pressing boards, so in order to introduce as few variables as possible, it is better compare the performance of the untreated wheat straw hot-pressing board and the dilute acid-pretreated wheat straw hot-pressing board, and highlight the necessity of dilute acid pretreatment. The hot-pressing process conditions (15 MPa, 2 h, and 160 °C) optimized with tensile strength as the performance index were selected.

**Figure 2:**
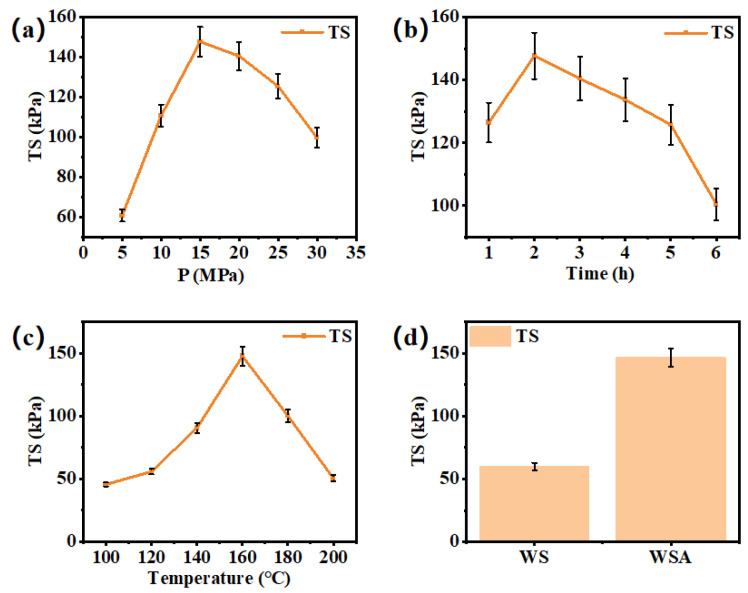
Tensile strength of boards under optimized hot-pressing conditions. (**a**) Pressure as the variable. (**b**) Temperature as the variable. (**c**) Time as the variable. (**d**) Blank control versus optimal hot-pressing conditions.

The tensile strength of wheat straw boards treated with dilute acid under varying hot-pressing conditions is presented in Figure 2a–c. Figure 2a shows the tensile strength of the board when the hot-pressing pressure is the variable, and the hot-pressing temperature is 160 °C and the hot-pressing time is 2 h. The application of hot-pressing pressure plays a crucial role in determining the thermoplastic flow and the degree of physical compaction among wheat straw fibers. Consequently, this pressure significantly impacts the density of the boards and the adhesive strength of the self-bonding, thereby influencing their physical and mechanical characteristics. If the hot-pressing pressure is insufficient, it fails to adequately compact the wheat straw, leading to uneven board density distribution and a reduced contact area among the straw particles. This results in a less dense internal structure and, consequently, decreased tensile strength. Conversely, excessive hot-pressing pressure can cause fiber breakage and overcompression of the wheat straw, rendering the boards brittle and ultimately compromising their tensile strength [23]. The optimal operating pressure determined through this project is 15 MPa. 

Figure 2b shows the tensile strength of the board when the hot-pressing time is the variable, and the hot-pressing temperature is 160 °C and the hot-pressing pressure is 15 MPa. The hot-pressing time significantly impacts the chemical structure and self-adhesive bonding force of wheat straw boards by influencing the thermoplastic deformation and moisture evaporation between the fibers. Consequently, it determines the physical and mechanical properties of the boards. If the hot-pressing time is insufficient, the lignin and cellulose in the dilute acid-pretreated wheat straw remain incompletely softened, resulting in inadequate bonding force between the fibers and, consequently, reduced tensile strength. Conversely, excessive hot-pressing time leads to excessive lignin flow within the wheat straw, decreasing effective bonding points and causing excessive evaporation of internal moisture. This increase in internal stress further compromises the tensile strength of the boards [24]. The optimal operating time obtained in this project is 2 h.

Figure 2c shows the tensile strength of the board when the hot-pressing temperature is the variable, and the hot-pressing time is 2 h and the hot-pressing pressure is 15 MPa. The hot-pressing temperature plays a crucial role in determining the physical and mechanical properties of wheat straw boards by influencing the thermoplastic behavior of cellulose and lignin. If the temperature is too low, lignin fails to soften adequately, and the hydrogen bonds among cellulose molecules remain intact, preventing effective bonding. Additionally, incomplete evaporation of moisture within the wheat straw hinders self-adhesion between fibers, leading to reduced mechanical strength. Conversely, excessively high hot-pressing temperatures can result in pyrolysis of cellulose, hemicellulose, and lignin, generating ash that disrupts self-adhesion and similarly compromises the mechanical strength of the boards [25]. The optimal operating temperature obtained in this project is 160 °C.

By meticulously optimizing the hot-pressing process conditions for dilute acid-treated wheat straw boards, it was discovered that the optimal parameters were 15 MPa pressure, 2 h duration, and 160 °C temperature. Under these optimal conditions, the dilute acid-pretreated wheat straw boards exhibited superior tensile strength compared to the untreated control group, as evident in Figure 2d. Dilute acid pretreatment played a pivotal role in enhancing the tensile performance of the boards. It effectively removed ash content, reducing the straw’s surface rigidity and facilitating compression molding. Additionally, the pretreatment partially decomposed hemicellulose, revealing functional groups on the cellulose surface, crucial for chemical bonding during hot-pressing. This led to stronger self-adhesive properties. Furthermore, dilute acid treatment disrupted some of the cellulose’s crystalline regions, promoting the rearrangement and recrystallization of amorphous regions. This process resulted in increased cellulose crystallinity. Although dilute acid pretreatment effectively improved the properties of wheat straw hot-pressed boards, compared with other commercial biomass boards, the tensile performance of wheat straw boards with dilute acid pretreatment still had significant room for improvement.

For the data shown in Figure 2, in the optimization part of operation parameters of the wheat straw board, we conducted a total of 16 groups of experiments to optimize temperature, pressure, and time for the hot-pressing board of wheat straw pretreated with dilute acid (in fact, three repeated experiments were carried out for each data point, and the error bar is shown in Figure 2; thus, a total of 48 experiments were run). Certainly, while TS remains a crucial metric, the evaluation of biomass-based sheets often encompasses bending strength and water absorption as well. However, given their significance in applications like furniture and building materials, it is imperative to prioritize the optimization of additional performance factors beyond mere TS strength. The aim of this project is mainly to improve the application scenario of wheat straw preparation board as furniture and other furniture materials through pretreatment and surface modification, and among these parameters, tensile strength is undoubtedly very important. Further optimization of other properties (such as bending strength and water absorption) only makes sense if there is a certain improvement in tensile strength.

We also realized that this experiment was a single point of optimization. With the tensile strength as the single optimization index of the operation parameter optimization process being slightly rough, and the prepared boards compared with the sizing and wood as raw materials, the performance was much worse, but this study focused on the study of coupling dilute acid pretreatment and surface modification treatment of wheat straw hot-pressing boards. Thus, only the optimization with tensile strength as a single index was carried out. Therefore, further modifications to the surface of these pretreated boards are necessary to elevate their performance.

### 3.2. Effects of Surface Modification on the Mechanical Properties of Dilute Acid-Pretreated Wheat Straw Boards

After dilute acid pretreatment and surface modification, the chemical structure of wheat straw would be changed, and the optimal hot-pressing process conditions (temperature, time, and pressure) would be different from the untreated hot-pressing process conditions. However, the purpose of this study is to investigate the effects of coupling dilute acid pretreatment and surface modification treatment on the properties of wheat straw hot-pressing board, so in order to introduce as few variables as possible and better compare the properties of the dilute acid pretreatment wheat straw hot-pressing board and the coupling dilute acid pretreatment and surface modification treatment wheat straw hot-pressing board, we highlight the necessity of coupling dilute acid pretreatment and surface modification treatment. The hot-pressing process conditions (15 MPa, 2 h, and 160 °C) optimized with TS as the performance index were selected.

**Figure 3:**
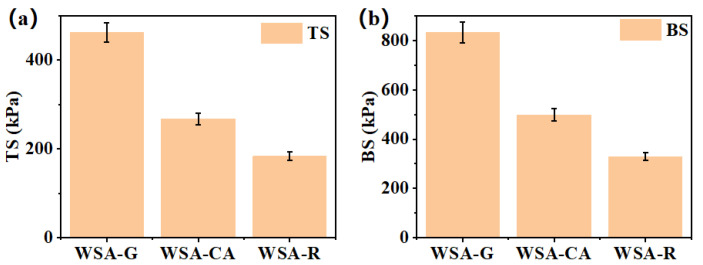
Mechanical strength of hot-pressed boards after surface modification of dilute acid-pretreated wheat straw. (**a**) Tensile strength (TS). (**b**) Bending strength (BS).

Surface modification treatments were applied to dilute acid-pretreated wheat straw, and the resulting mechanical strengths are presented in Figure 3a,b. Notably, glutaraldehyde exhibited the most effective surface modification among the tested agents. Wheat straw glutaraldehyde pretreatment samples demonstrated the highest tensile strength (TS) and bending strength (BS), reaching 463 kPa and 833 kPa, respectively. Citric acid followed closely, achieving a tensile strength of 267 kPa and a bending strength of 498 kPa. Rosin, on the other hand, had the least significant impact on surface modification, with a tensile strength of 183 kPa and a bending strength of 329 kPa. The superior performance of glutaraldehyde can be attributed to its aldehyde groups, which react with the exposed hydroxyl groups on the cellulose of dilute acid-pretreated wheat straw. This reaction creates a crosslinked network that significantly enhances the mechanical properties of the boards. Citric acid also improves board performance by forming a crosslinked network through esterification reactions between its carboxyl groups and the exposed hydroxyl groups on the cellulose. Rosin, on the other hand, enhances board performance primarily through physical adsorption on the surface of wheat straw fibers and esterification reactions with the hydroxyl groups exposed by the dilute acid pretreatment. The remarkable difference in tensile strength observed between glutaraldehyde-modified samples and the other two groups can be primarily attributed to the highly crosslinked network formed by the etherification reaction between glutaraldehyde and dilute acid-pretreated wheat straw.

Compared to the mechanical strength of straw boards prepared by other methods without adhesive [22,26,27,28,29], there remains a discernible gap in the mechanical properties of wheat straw boards prepared through the combined dilute acid pretreatment and surface modification. Nevertheless, the present study shows that the integration of these two processes can significantly improve the mechanical strength of wheat straw boards without the need for adhesives. This offers a promising alternative method for the preparation of adhesive-free boards, providing a new benchmark for sustainable and environmentally friendly board production.

### 3.3. Analysis of Chemical Structure of Wheat Straw Board with Surface Modified Dilute Acid Pretreatment

**Figure 4:**
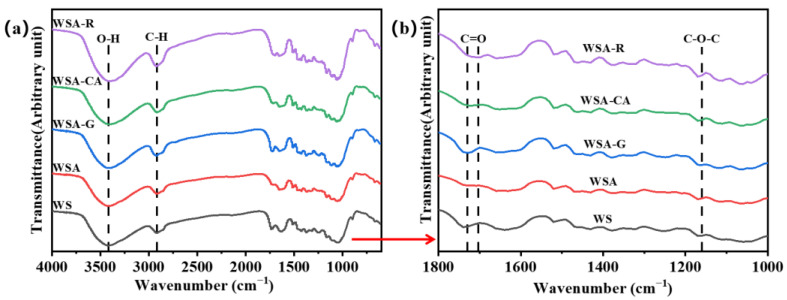
FTIR spectra of dilute acid-pretreated wheat straw boards with different surface modifications. (**a**) Wavenumber 600–4000 cm^−1^; (**b**) Wavenumber 1000–1800 cm^−1^.

FTIR characterization was employed to gain insights into the functional groups of the materials. The resulting FTIR spectra of wheat straw boards pretreated with dilute acid and further surface-modified in various ways are presented in Figure 4. All five samples exhibited a broad peak at 3400 cm^−1^, indicative of O-H stretching vibrations, confirming the presence of O-H bonds, a typical characteristic of alcohols and phenols, representing the abundant cellulose component in wheat straw. A peak at 2918 cm^−1^ indicates the stretching vibrations of C-H bonds in alkanes, caused by the vibrations of C-H bonds in the alkyl chains of hemicellulose. A sharp peak at 1162 cm^−1^ suggests the presence of C-O-C ether bonds, indicating etherification reactions between the aldehyde groups contained in glutaraldehyde and the hydroxyl groups of dilute acid-pretreated wheat straw. Peaks between 1679 cm^−1^ and 1730 cm^−1^ are related to the stretching vibrations of C=O carbonyl double bonds; the peak at 1679 cm^−1^ in wheat straw indicates the presence of proteins (amide bonds) or other carbonyl-containing compounds, while the peak at 1730 cm^−1^ represents the esterification reaction between citric acid, rosin, and the hydroxyl groups of dilute acid-pretreated wheat straw, forming characteristic ester functional groups. Compared with wheat straw samples, the peak strength reduction in wheat straw acid pretreatment samples at 1730 cm^−1^ and 1679 cm^−1^ is related to the removal of hemicellulose by dilute acid pretreatment. Spectral analysis reveals that after dilute acid pretreatment of wheat straw, part of the hemicellulose is removed, exposing functional groups on the cellulose surface, which is beneficial for enhancing board performance and further surface modification. After modification, the -O- ether bond at 1162 cm^−1^ proves the etherification reaction between the aldehyde groups in the glutaraldehyde modifier and the hydroxyl groups of dilute acid-pretreated wheat straw. In contrast, the ester group at 1730 cm^−1^ proves the esterification reaction between the carboxyl groups in the citric acid, rosin modifiers, and the hydroxyl groups of dilute acid-pretreated wheat straw, both enhancing board performance.

### 3.4. Surface Morphology Analysis of Modified Dilute Acid Pretreatment Wheat Straw Boards

**Figure 5:**
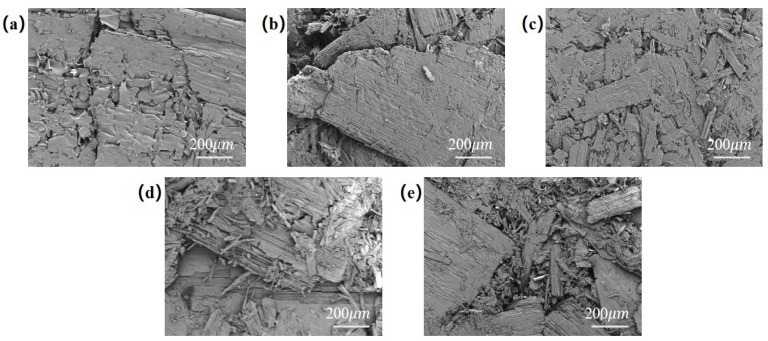
Surface microstructure of dilute acid-pretreated wheat straw boards with different surface modifications. (**a**) Wheat straw sample. (**b**) Wheat straw acid pretreatment sample. (**c**) Wheat straw glutaraldehyde pretreatment sample. (**d**) Wheat straw citric acid pretreatment sample. (**e**) Wheat straw rosin pretreatment sample.

The microstructural analysis of dilute acid-pretreated wheat straw boards with varying surface modifications is presented in Figure 5. As observed in Figure 5a, the wheat straw sample maintains its natural roughness with an uneven surface texture and numerous irregular pores. In contrast, the wheat straw acid pretreatment sample shown in Figure 5b exhibits a smoother surface due to dilute acid pretreatment, revealing a looser fiber arrangement and increased porosity compared to the wheat straw sample. This is attributed to the partial dissolution of hemicellulose in wheat straw by the dilute acid. The wheat straw glutaraldehyde pretreatment sample depicted in Figure 5c demonstrates the effect of glutaraldehyde surface modification, resulting in a smoother and more uniform surface texture. The wheat straw fibers appear more bonded due to the crosslinking reaction of glutaraldehyde, promoting self-adhesion between the fibers and significantly reducing porosity. The wheat straw citric acid pretreatment sample in Figure 5d reveals a smoother and more uniform surface with less fragmentation of wheat straw fibers. Although pores are still present, they appear more uniform, indicating that citric acid enhances the adhesiveness between the fibers. Finally, the wheat straw rosin pretreatment sample shown in Figure 5e exhibits a distinct coating on the board surface after rosin surface modification. This modification reduces porosity compared to the wheat straw sample, indicating that rosin fills some of the gaps between wheat straw fibers, resulting in a tighter fiber arrangement. Overall, the micrograph analysis indicates that the combination of dilute acid pretreatment and surface modification significantly improves the uniformity and smoothness of the hot-pressed wheat straw board surface, thereby enhancing its performance.

### 3.5. Thermogravimetric Analysis of Surface-Modified, Dilute Acid-Pretreated Wheat Straw Boards

**Figure 6:**
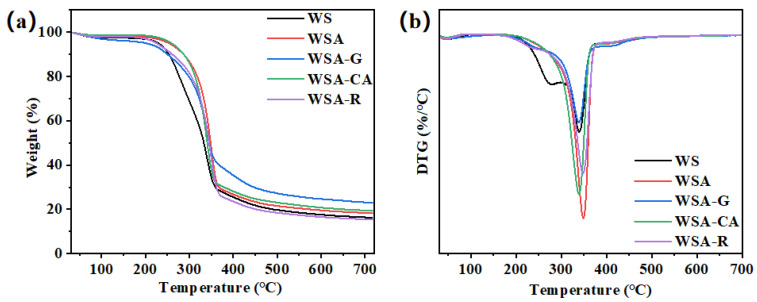
Thermogravimetric analysis of dilute acid-pretreated wheat straw boards with different surface modifications. (**a**) Thermogravimetry analysis of dilute acid-pretreated wheat straw boards with various surface modifications. (**b**) Differential thermogravimetry analysis of dilute acid-pretreated wheat straw boards with different surface modifications.

Thermogravimetric analysis serves as a valuable tool for assessing the thermal stability and decomposition performance of materials. In Figure 6, we present the thermogravimetric analysis of dilute acid-pretreated wheat straw boards, subjected to various surface modifications. These boards exhibit remarkable thermal stability and resistance to degradation during combustion. The mass loss observed in a nitrogen-purged closed environment reflects a singular stage of thermal decomposition. As Figure 6a illustrates, a pronounced reduction in thermal degradation occurs between 250 °C and 365 °C. This decrement is attributed to the thermal breakdown of lignin, cellulose, and hemicellulose components present within the board samples. Notably, the distinct surface modifications applied to the wheat straw boards do not seem to significantly alter the overall thermal decomposition pattern. This suggests that the thermal stability of these boards is primarily governed by their chemical composition and the effects of dilute acid pretreatment, rather than the specific surface modifications employed. The findings from this thermogravimetric analysis provide valuable insights into the thermal behavior of dilute acid-pretreated wheat straw boards, which can inform their potential applications and durability in various settings [30]. Analysis reveals that surface-modified, dilute acid-pretreated wheat straw boards exhibit an elevated initial degradation temperature compared to untreated boards. This is attributed to the formation of a robust ether bond crosslinked structure through the etherification reaction between glutaraldehyde’s aldehyde groups and hydroxyl groups. Additionally, the presence of a more stable ester bond arises from the esterification reaction of citric acid with hydroxyl groups. Furthermore, the esterification reaction of rosin with hydroxyl groups forms ester bonds, which also act as a protective coating on the board surface, thus elevating the energy requirement for thermal decomposition. Among boards treated with various surface modifiers, glutaraldehyde surface-modified, dilute acid-pretreated wheat straw boards exhibit the lowest mass loss. Figure 6b depicts the differential thermogravimetry analysis, indicating that these boards possess a notably lower maximum degradation rate compared to those prepared with alternative surface modifiers. This observation underscores the superior thermal stability and heat degradation resistance of glutaraldehyde-modified boards during combustion.

The thermogravimetric analysis further demonstrates that the thermal stability of hot-pressed wheat straw boards undergoes significant enhancement after dilute acid pretreatment. Notably, surface modification treatment further boosts their thermal stability and heat degradation resistance. These findings highlight the potential of surface-modified, dilute acid-pretreated wheat straw boards in applications demanding durability and thermal resilience.

### 3.6. Water Absorption Analysis of Surface-modified, Dilute Acid-Pretreated Wheat Straw Boards

**Figure 7:**
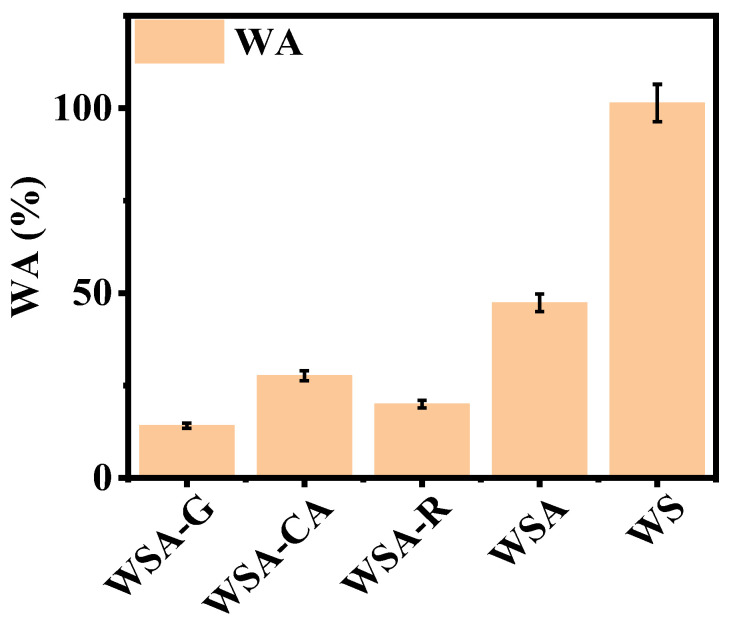
Water absorption rates of dilute acid-pretreated wheat straw boards with different surface modifications.

The water absorption rate serves as a crucial metric for evaluating the performance of boards. As depicted in Figure 7, a comparative analysis of water absorption rates was conducted on various surface-modified dilute acid-pretreated wheat straw boards. It is evident that the integration of dilute acid pretreatment with surface modification significantly reduces the water absorption rate of the boards, indicating a remarkable enhancement in their waterproofing capabilities. Among these boards, wheat straw glutaraldehyde pretreatment samples, which employed glutaraldehyde as the surface modifier, exhibited the lowest water absorption rate, indicating superior water resistance. This can be attributed to glutaraldehyde’s reaction with the hydroxyl groups exposed on the cellulose of the dilute acid-pretreated wheat straw, resulting in a more stable crosslinked structure. This crosslinked structure effectively reduces porosity on the board surface, thereby enhancing its waterproofing performance. On the other hand, wheat straw rosin pretreatment samples, prepared with rosin as the surface modifier, demonstrated the second-lowest water absorption rate. This is due to the esterification and polymerization reactions that occur between rosin and the dilute acid-pretreated wheat straw under hot-pressing conditions. These reactions form a resinous coating that covers the surface of wheat straw fibers, effectively improving the waterproofing properties of the boards. The analysis further reveals that the waterproofing characteristics of hot-pressed boards undergo a noticeable improvement after dilute acid pretreatment of wheat straw. Furthermore, the introduction of surface modification treatment further boosts their waterproofing performance, underscoring the importance of surface modification in enhancing the durability and performance of wheat straw boards.

### 3.7. Self-Bonding Mechanism of Surface-Modified, Dilute Acid-Pretreated Wheat Straw Boards

In contrast to conventional glued boards, the wheat straw hot-pressed boards developed in this study, through surface-modified dilute acid pretreatment, eliminate the need for adhesive addition. These boards exhibit exceptional mechanical strength and thermal resistance. 

**Figure 8:**
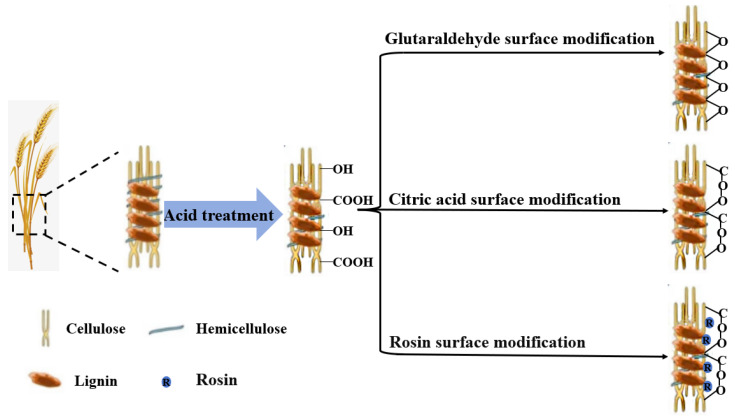
Schematic of the self-bonding mechanism for surface-modified, dilute acid-pretreated wheat straw boards.

Figure 8 illustrates the bonding mechanism behind their construction. During dilute acid pretreatment, wheat straw experiences partial removal of hemicellulose, enabling surfactants to react with exposed functional groups on wheat straw fibers, thus augmenting the boards’ self-adhesiveness. During the hot molding process, glutaraldehyde’s aldehyde groups react with the cellulose’s hydroxyl groups in the pretreated wheat straw, creating a crosslinked structure with stable ether bonds (-O-). This interaction enhances the self-adhesiveness among the fibers of the pretreated wheat straw. Moreover, citric acid, a multifunctional carboxylic acid, reacts with the exposed hydroxyl groups on the cellulose of the pretreated wheat straw, forming ester bonds (-COO-). This reaction raises the crosslinking density of the boards, further improving their mechanical properties. Rosin, rich in conjugated double bonds, carboxyl groups, and other unsaturated bonds, undergoes esterification and polymerization reactions during hot-pressing. These reactions generate ester bonds (-COO-) and a resinous coating that physically binds between the board fibers, significantly boosting their self-adhesive capabilities.

In summary, the wheat straw hot-pressed boards developed in this study combine innovative surface modification and dilute acid pretreatment to achieve exceptional mechanical and thermal resistance properties, eliminating the need for additional adhesives and enhancing their overall performance.

## 4. Conclusions

This study was dedicated to exploring the preparation of hot-pressed wheat straw boards, with a particular emphasis on analyzing the individual and combined impacts of dilute acid pretreatment and subsequent surface modification.

(1) This work determined the optimal hot-pressing conditions, revealing that the boards prepared using dilute acid-pretreated wheat straw exhibited the best performance when hot-pressed at a temperature of 160 °C, under a pressure of 15 MPa, and for a duration of 2 h.
(2) Under the optimized hot-pressing parameters, the wheat straw boards treated with dilute acid pretreatment exhibited a significant increase in tensile strength compared to the untreated control group. This treatment eliminated the ash content from the wheat straw, decreasing its surface rigidity and facilitating compression molding. Additionally, the dilute acid pretreatment effectively decomposed hemicellulose, revealing the functional groups on the cellulose surface, enabling the formation of stronger chemical bonds during hot-pressing, thereby bolstering the boards’ self-adhesiveness.
(3) Under the optimal hot-pressing conditions, the performance of wheat straw boards that undergo both surface modification and dilute acid pretreatment significantly surpasses those prepared with dilute acid pretreatment alone or untreated wheat straw. This coupling of processes leads to boards with superior mechanical properties, making them more suitable for various applications. Among them, boards treated with glutaraldehyde exhibited the most significant enhancement, achieving a tensile strength of 463 kPa, bending strength of 833 kPa, and a water absorption rate of 14.14%.

In conclusion, this study underscores the significant potential of coupling dilute acid pretreatment with surface modification to enhance the mechanical properties of wheat straw boards. By pretreating wheat straw to prepare nonadhesive hot-pressing boards, the significance of biomass conversion difference is reflected, compared with direct burning of wheat straw. Firstly, wheat straw was pretreated to prepare nonadhesive hot-pressed boards, which effectively improved the utilization rate of biomass resources. Secondly, the use of nonadhesive technologies, especially those relying on biomass conversion, can effectively protect the environment and promote sustainable development. This study used hydrochloric acid as a reagent for acid pretreatment, and, in fact, according to the ideas presented in this paper, hydrochloric acid can be replaced with inorganic acids such as sulfuric acid and nitric acid, or organic acids such as formic acid and acetic acid. In addition, the glutaraldehyde modifier used in this study can be replaced with formaldehyde, and rosin modifier can be replaced with paraffin wax. Although, compared to the mechanical strength of commercial boards, there remains a discernible gap in the mechanical properties of wheat straw boards prepared through the combined dilute acid pretreatment and surface modification, this study mainly provides a preparation idea and method based on the pretreatment of agricultural solid waste wood fiber raw materials for hot-pressed boards. Dilute acid treatment can remove some hemicellulose and impurities in wheat straw, fully exposing the functional groups on wheat straw cellulose, indicating that the composition of raw materials can greatly affect the conversion of downstream chemicals or functional materials. Subsequent research can develop more environmentally friendly and more efficient wood fiber solid waste pretreatment and surface modification technologies, innovate the preparation technology of wood fiber biomass boards, and promote sustainable development.

## Data Availability

Data are contained within the article.
